# Calcifications of the Thoracic Aorta on Extended Non-Contrast-Enhanced Cardiac CT

**DOI:** 10.1371/journal.pone.0109584

**Published:** 2014-10-10

**Authors:** Damian Craiem, Gilles Chironi, Mariano E. Casciaro, Sebastian Graf, Alain Simon

**Affiliations:** 1 Favaloro University. Facultad de Ingeniería, Ciencias Exactas y Naturales, CONICET, Buenos Aires, Argentina; 2 APHP, Hôpital Européen Georges Pompidou, INSERM U970, Université Paris-Descartes, Paris, France; University Medical Center (UMC) Utrecht, Netherlands

## Abstract

**Background:**

The presence of calcified atherosclerosis in different vascular beds has been associated with a higher risk of mortality. Thoracic aorta calcium (TAC) can be assessed from computed tomography (CT) scans, originally aimed at coronary artery calcium (CAC) assessment. CAC screening improves cardiovascular risk prediction, beyond standard risk assessment, whereas TAC performance remains controversial. However, the curvilinear portion of the thoracic aorta (TA), that includes the aortic arch, is systematically excluded from TAC analysis. We investigated the prevalence and spatial distribution of TAC all along the TA, to see how those segments that remain invisible in standard TA evaluation were affected.

**Methods and Results:**

A total of 970 patients (77% men) underwent extended non-contrast cardiac CT scans including the aortic arch. An automated algorithm was designed to extract the vessel centerline and to estimate the vessel diameter in perpendicular planes. Then, calcifications were quantified using the Agatston score and associated with the corresponding thoracic aorta segment. The aortic arch and the proximal descending aorta, “invisible” in routine CAC screening, appeared as two vulnerable sites concentrating 60% of almost 11000 calcifications. The aortic arch was the most affected segment per cm length. Using the extended measurement method, TAC prevalence doubled from 31% to 64%, meaning that 52% of patients would escape detection with a standard scan. In a stratified analysis for CAC and/or TAC assessment, 111 subjects (46% women) were exclusively identified with the enlarged scan.

**Conclusions:**

Calcium screening in the TA revealed that the aortic arch and the proximal descending aorta, hidden in standard TA evaluations, concentrated most of the calcifications. Middle-aged women were more prone to have calcifications in those hidden portions and became candidates for reclassification.

## Introduction

Calcified atherosclerosis has been associated with a higher risk of mortality but the strength of this relation depends on the affected vascular beds [1]. In particular, thoracic aorta calcium (TAC) has been related to all-cause mortality, independently of conventional cardiovascular risk factors and the presence of coronary artery calcium (CAC) [2]. TAC is less frequently performed than CAC and its ability to further refine clinical event prediction is still controversial [3], [4]. A recent report states that CAC but not TAC measurements improve cardiovascular risk prediction beyond standard risk assessment [5]. Other reports indicate that TAC is a better predictor of future coronary events than CAC only in women [6] or when analyzing non-cardiac events [7]. Unfortunately, as the TAC score is commonly calculated on the same CT coronary scan of CAC studies, only the ascending and descending aortic portions are partially visible and the aortic arch is systematically excluded. Eventually, this lack of consistency to determine the exact parts of the TA included in TAC screening may have limited prior assessments for the prognostic value of TAC. The curvilinear portion of the aorta has a complex geometry that demands advanced segmentation algorithms to properly investigate its morphology [8], [9]. An extended scan length might improve not only the detection of lesions associated with the risk of ischemic stroke, but also the early recognition of aneurysms [10], [11]. In this study, we investigated the differences in TAC assessment using an extended CT scan that included the aortic arch, with respect to a standard heart scan. In particular, patients that would escape calcium detection with a standard measurement method were stratified and analyzed separately as candidates for reclassification.

## Methods

### Study Subjects

Individuals were initially referred to our cardiovascular prevention unit by occupational health physicians or general interns for in-depth stratification of coronary risk. In the present study we included all patients over 2 years from September 2009 who had undergone extended (including the aortic arch) non-contrast cardiac computed tomography (CT) for CAC and aortic aneurisms screening during their hospitalization. For this study, only primary prevention patients without symptoms were included. Weight and height were measured for calculation of body mass index. Brachial blood pressure (BP) was determined as the mean of 3 measurements by sphygmomanometer procedure in the supine position after a 10 minute rest. Hypertension was defined by BP of 140/90 mmHg or above, or use of anti-hypertensive medication. Total and HDL (high-density lipoprotein) blood cholesterol and triglyceride were measured in the supine position after 14 hours fasting and LDL (low-density lipoprotein) was calculated with the Friedewald formula or, if not applicable, directly measured. Hypercholesterolemia was defined by fasting LDL cholesterol above 3.3 mmol/l or by presence of LDL-lowering drug treatment. Blood glucose was measured after an overnight fast and diabetes was defined by fasting blood glucose of 7 mmol/l or above, or by presence of antidiabetic medication.

The retrospective analysis of personal health data of study subjects had the authorization of the CNIL (*Commission nationale de l'informatique et des libertés*) and was in accordance with the Helsinki declaration. Patient information was anonymized and de-identified prior to analysis.

### Image acquisition

Aortic images were obtained with non-contrast cardiac 64-slice MSCT (Light-speed VCT; GE Health care, Milwaukee, Wisconsin, USA) during an extended scan length acquisition. Images were acquired with prospective-ECG gating at 60% of R-R interval in the cranio-caudal direction from the top of the aortic arch to the level of the diaphragm. Measurements were taken with 2.5 mm axial slices, 120 kVp, 250-mA tube current, 250-ms exposure time, and average 250-mm field of view. Scans were analyzed by custom-designed software that estimated the thoracic aorta (TA) geometry in 3D and quantified the TA calcifications. The effective radiation dose was assessed in a group of 200 patients and was 1.23±0.14 mSv (range 0.96–2.1 mSv) [8].

### Semiautomatic segmentation algorithm

**Figure 1:**
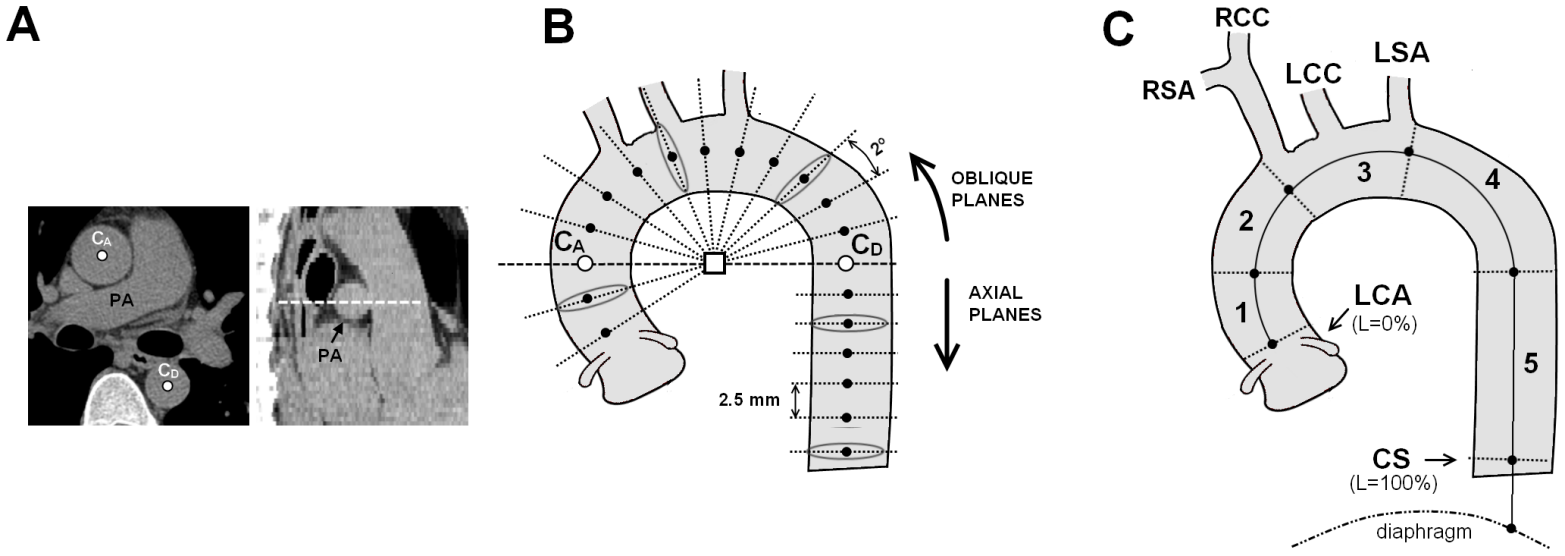
Thoracic aorta segmentation. **A**. The user selected 2 seed points in the center of ascending (C_A_) and descending (C_D_) aorta at the level of the pulmonary artery bifurcation. **B**. The algorithm sequentially inscribed circles inside the vessel cross-section using axial planes below C_D_ and oblique planes above for the curvilinear portion. **C**. The vessel was divided into 5 segments using anatomical landmarks. LCA: left coronary artery. RSA: Right subclavian artery. RCC and LCC: right and left carotid arteries. LSA: left subclavian artery. CS: coronary sinus.

Details of the complete segmentation process to separate the TA from surrounding tissues and to analyze its 3D geometry were recently reported [8], [12]. For each patient, the custom software isolated the thoracic aorta, assessed its geometry in 3D and quantified the calcifications. Briefly, the user started with a manual selection of two seed points in the axial slices at the central level of the pulmonary artery (see C_A_ and C_D_ in Figure 1A). Then, an automatic algorithm extracted the central skeleton and estimated the vessel diameter, dynamically expanding and centering circles to inscribe them inside the vessel cross-section area [8]. This circle-fitting algorithm was sequentially applied over the axial CT slices for the descending portion of the aorta and over oblique planes for the curvilinear part (Figure 1B). These planes were reconstructed in steps of 2° angles by trilinear interpolation following a semitoroidal path until an angle of 240°. In both cases, the center point of the circle found at the end of the algorithm was used as a seed point for the next slice or reconstructed plane. As the curvilinear portion of the aorta did not necessarily follow a strict toroidal shape and the descending aorta was not perfectly vertical, a post-processing correction was performed to ensure that reconstructed planes remained perpendicular to the true aortic centerline. The result of this process in each patient was a list of ≈150 centerline points with the corresponding diameters that approximated the cross section of the aorta in each position.

To finish the TA geometry assessment, the user indicated 4 anatomic landmarks: Left main coronary artery (LMCA), Right and left subclavian arteries (RSA and LSA) and the coronary sinus (CS). The aorta was then divided in 5 segments as shown in Figure 1C. The whole automated process was reported to take less than 1 min per patient, with maximum intra- and inter-observer coefficient of variation values of 0.4% for TA diameter and 4.4% for TA volume [8].

### Calcification assessment

Lesions were quantified with a semi-automatic algorithm using the Agatston score method [13]. For each axial image, the algorithm highlighted in red all candidate lesions. Then, the user went over every axial plane and clicked on the red lesions to validate them and turn them green. At this point, the Agatston score was automatically calculated for each green lesion that was then orthogonally projected over the vessel centerline curve and associated with the corresponding segment (from 1 to 5). Finally, the lesions scores were accumulated for each segment. Global and segmental scores were reported for each subject.

### Candidates for reclassification

Patients with calcifications that would escape detection with a standard scan (either CAC or TAC), were designated as candidates for reclassification. For such stratified analysis, the population was divided into 3 groups. Group 1: patients free of calcifications (CAC = 0 and TAC = 0). Group 2: patients detected with the standard method (CAC>0 or standard TAC>0). Group 3: patients detected exclusively with the extended measurement method (CAC = 0 and standard TAC = 0 and extended TAC>0). The characteristics of each group were analyzed separately. Ten-year Framingham risk score (10-years FRS, % probability of CHD event in the next 10 years) was estimated with the Framingham risk model by entering age, total cholesterol, high-density lipoprotein (HDL) cholesterol, and systolic blood pressure as continuous variables and sex, diabetes, and current smoking as categorical variables (presence or absence) [14]. Comparisons for age and FRS between the 3 groups were made with ANOVA and Tukey's HSD post hoc test. We used chi-square test for categorical data and post hoc tests were performed pair-wise with a Bonferroni correction for multiple comparisons (e.g. 3). A value of p<0.05 was considered significant.

## Results

Table 1 shows the baseline characteristics of the study population (n = 970). Presence and extent of CAC, standard assessed TAC and extended TAC are reported in Table 2. Standard TAC was calculated only including lesions in segments 1 and 5, whereas extended TAC included all 5 segments. Overall, 62% and 64% of subjects had detectable CAC and TAC, respectively. TAC prevalence would drop to 31% using a standard scan. Almost half of the patients had both CAC and TAC whereas 22% did not have any detectable calcium.

**Table 1:** Population description. Continuous variables are expressed as mean±SD.

Characteristics	
**Number of subjects, n**	970
**Male gender, n (%)**	754 (77)
**Age, yrs**	57±9
**Body mass index, kg.m^−2^**	26.4±4.2
**Hypertension, n (%)**	476 (49)
**Hypercholesterolemia, n (%)**	796 (82)
**Current or past smokers, n (%)**	524 (54)
**Diabetes, n (%)**	83 (9)
**10-years Framingham risk score, %**	10±6

**Table 2:** Presence and extent of coronary artery and thoracic aorta calcifications. Presence/absence of calcium and Agatston scores are reported.

Calcifications	
Extended TAC >0, n (%)	618 (64)
Extended TAC score, median [25–75 percentile]	39 [0–333]
Standard TAC >0, n (%)	296 (31)
Standard TAC score, median [25–75 percentile]	0 [0–5]
CAC >0, n (%)	598 (62)
CAC score, median [25–75 percentile]	16 [0–148]
CAC >0 and extended TAC >0, n (%)	457 (47)
CAC = 0 and extended TAC = 0, n (%)	211 (22)

Standard TAC was calculated only including segments 1 and 5. Extended TAC included all 5 segments as in Figure 1C.

**Figure 2:**
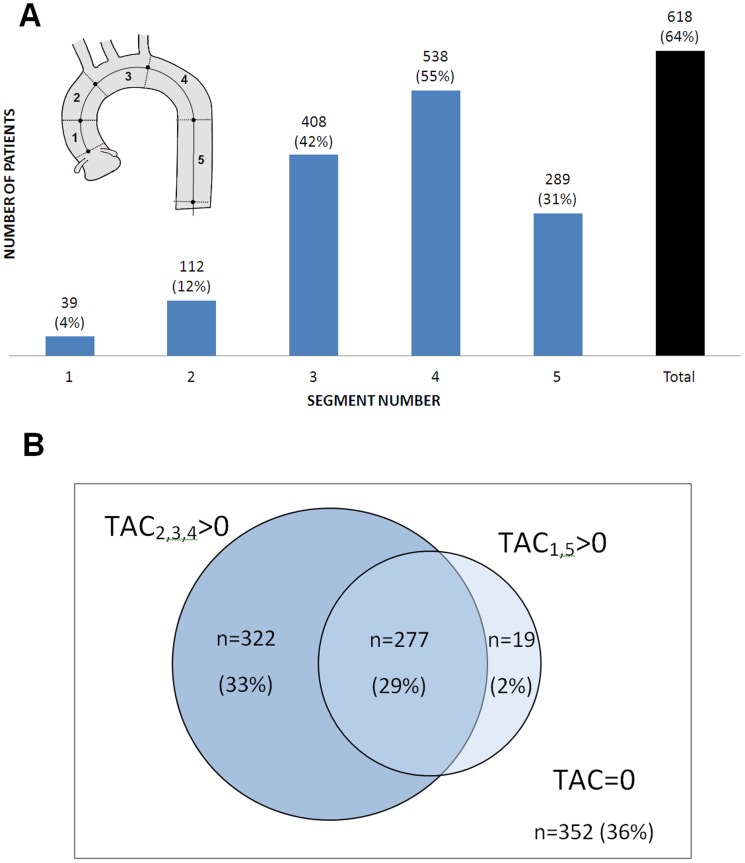
Calcified thoracic aorta segments in the whole population (n = 970). **A**. Number and proportion of patients with TAC score>0 by aortic segments. **B**. Number and percentage of patients with calcifications assessed with the standard method that explored segments 1 and 5 (TAC_1,5_>0) and the extended method that included the aortic arch (TAC_2,3,4_>0).

TAC prevalence was heterogeneous across the aortic segments as shown in Fig 2A. Calcifications appeared in 4%-to-12% of subjects in the ascending portions, increased to 42% in the arch and attained a peak of 55% in the proximal descending segment. Distally, TAC was detected in 31% of subjects. The impact of exploring segments 2, 3 and 4 in the extended method with respect to a standard TA scan is shown in Figure 2B. Of 64% of subjects that had TAC, 33% were detected exclusively with the extended method. Only 2% of subjects had TAC in segments 1 and/or 5 and not in segments 2, 3 or 4. Both methods would identify the other 29% of subjects with calcifications. The inspection of segments 2, 3 and 4 would ensure the detection of 97% of patients with aortic calcifications.

**Figure 3:**
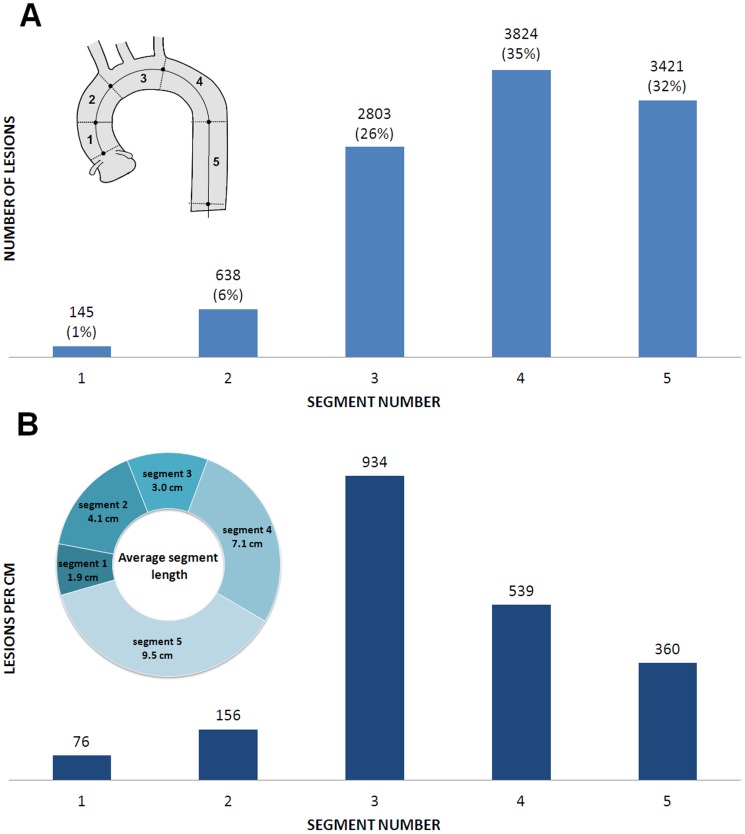
Spatial distribution of 10831 calcifications found in 618 patients across thoracic aorta segments. **A**. Number of calcifications by segment and % of total number of calcifications. **B**. Number of calcifications by average segment length in cm.

The distribution of calcifications across the aortic segments is shown in Figure 3A. We quantified 10831 calcifications in 618 subjects. Most of the lesions were found in the proximal descending portion (n = 3824 lesions, 35%), followed by descending aorta (n = 3421, 32%) and the aortic arch (n = 2803, 26%). Only 7% of the total number of calcifications was found in the ascending aorta (segments 1 and 2). When the number of calcifications was expressed by unit of segment length, the aortic arch appeared as the most diseased with 934 calcifications per cm (Fig 3B).

**Figure 4:**
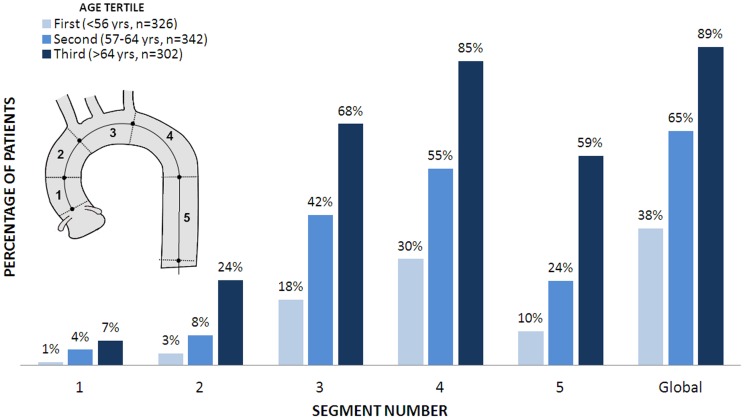
Proportion of patients with calcifications across thoracic aorta segments separated by age tertiles.

The prevalence of TAC by age tertiles for the 5 segments is shown in Figure 4. Globally, we found a 38% of prevalence for the 1^st^ tertile of age, 65% for the 2^nd^ and 89% for the 3^rd^ tertile. The proximal descending aorta was affected in 85% of subjects above the 3^rd^ tertile of age.

### Reclassification candidates

To discover whether reclassification of subjects that escaped detection by the standard method is possible, participants were divided into 3 groups according to the presence of calcium in the coronary arteries and/or in the TA. Overall, 22% of subjects were free of any calcium (Group 1), 67% had either CAC and/or TAC detectable with a standard scan (Group 2) and 11% were exclusively found with the extended measurement method (Group 3). Characteristics of each group are shown in Table 3. Age and 10-years FRS were significantly different between groups (p<0.001, ANOVA). A higher proportion of women was observed in Group 3 compared to the other groups (p<0.05). Risk scores did not differ between Groups 1 and 3.

**Table 3:** Stratified analysis to identify candidate patients for reclassification.

Characteristics	GROUP 1 (no calcium)	GROUP 2 (standard method)	GROUP 3 (reclassification candidates)	P value
Number of subjects, n (%)	211 (22)	648 (67)	111 (11)	
Male gender, n (%)	167 (79)	527 (81)	60 (54)* +	<0.001
Age, yrs	50±8	59±8*	55±8* +	<0.001
Hypertension, n (%)	80 (38)	354 (55)*	42 (38)+	<0.001
Hypercholesterolemia, n (%)	146 (69)	561 (87)*	89 (80)	<0.001
Diabetes, n (%)	19 (9)	54 (8)	10 (9)	NS
Current or past smokers, n (%)	104 (49)	371 (57)*	49 (44)+	<0.05
10-years Framingham risk score, %	8±5	11±6*	8±5+	<0.001

Group 1: CAC = 0 and extended TAC = 0 (free of calcium). Group 2: CAC>0 or standard TAC>0. Group 3: CAC = 0 and standard TAC = 0 and extended TAC>0. CAC = Coronary artery calcium. Standard TAC was calculated only including segments 1 and 5. Extended TAC included all 5 segments (see Figure 1C).

*p<0.05 with respect to Group1.

+ p<0.05 with respect to Group 2.

Patients were separated into subjects free of calcium (Group 1), those assessed with traditionally measured method (Group 2) and candidates for reclassifications that were exclusively assesed with the proposed extended method (Group 3).

## Discussion

In this study we show that calcified lesions in the TA of asymptomatic patients occurred at two preferential sites that remain “invisible” in routine CAC screening: the aortic arch and the proximal descending aorta. These hidden regions were explored by extending the scan length of a standard coronary scan to cover the aortic arch. Using the enlarged scan, TAC prevalence doubled from 31% to 64%. After accurately reading 970 heart scans, 60% of the almost 11000 calcifications were identified in the upper segments, the aortic arch being the most densely affected sector per cm length. To our knowledge, this is the first geometrical TA study that has additionally calculated the TAC Agatston score, including the aortic arch, in a cohort of almost 1000 asymptomatic patients.

The detection of coronary and aortic calcification in our population was 62% and 64%, respectively. In other reports that did not include the aortic arch, these percentages averaged 47%-to-56% and 22%-to-31%, respectively [2], [3], [6], [15], [16]. Certainly, the extension of the scan length increased the TAC prevalence detected. In fact, if we only consider segments 1 and 5, our values did not differ from others. Reports where all aortic segments were measured are scarce. Jacobs et al. measured the whole TA in a sub-cohort of 958 asymptomatic subjects (from a population of 7557 heavy smokers) and found a prevalence of CAC and TAC above 60%, closer to our values [7]. It is to note that the proportions of men, hypertensive and hypercholesterolemic patients were also comparable with our study. Allison et al. studied 4544 patients that underwent a whole body CT scanning and found a 55% and 37% of prevalence for CAC and TAC, respectively [1]. Probably, TAC prevalence detected was lower than in our study due to a higher participation of women and a healthier cohort. Direct comparisons with other studies should be made with caution, because small differences associated with the population risk profile might introduce substantial bias in prevalence values [17].

Essentially, the main finding of our work is that about half of patients with no visible TAC on a standard coronary scan were detected with the extended method. To search for patients that could be reclassified, we performed a stratified analysis in three groups, including coronary and/or thoracic calcifications and comparing both methods. We found 11% of subjects that would escape any calcium detection with a standard scan (Group 3). It is noteworthy that globally, 33% of subjects would be considered free of calcium using the standard TA evaluation, instead of the true 22% reported for Group 1. Candidates for reclassification in Group 3 were 5 years older than patients with no calcium and 4 years younger than those detected with the standard method. We also observed a higher proportion of women in this group (46% vs ≈20% in the other 2 groups) and a risk profile closer to subjects free of calcium. This information suggests that middle-aged women at increased cardiovascular risk could benefit most from an extended scan for TAC detection and they would have a better chance to be reclassified.

The spatial distribution of calcifications through the aortic segments in our study was far from homogeneous. It revealed a high prevalence in the descending aorta and showed a predilection for the aortic arch when normalized per segment length. Calcifications were distributed as follows: 9% for the ascending segment, 25% for the arch and 66% for the descending aorta. Only a few previous studies analyzed the complete aorta and they globally identified the aortic arch as a vulnerable place for calcifications. Takasu et al. divided the TA into 6 segments and found calcifications predominantly in the aortic arch [18]. In another large Japanese cohort aimed at screening lung cancer, the frequency of calcification for the ascending was 3%, attained a peak of 20% at the aortic arch and dropped to 10% for the descending [19]. For the same three segments using transesophageal electrocardiography in a general population, Agmon et al. found percentages of 8%, 31% and 45%, respectively [20]. Possible explanations for this heterogeneous distribution remain somehow speculative and include disturbed flow and wall shear stress conditions [21], [22], small geometrical ondulations in the vecinity of ductus arteriosius scar and intercostal branches [23] and LDL concentration and transportation dynamics [24], [25].

A strong association between TAC and age was observed in the aortic arch and the descending aorta. A global prevalence of almost 90% was found for age>64years, being again the aortic arch and the descending aorta the most affected sites. The exponential increment of TAC prevalence with age, that even surpasses CAC after the age of 70 years, was reported before [1], [4]. Our global prevalence for older subjects seems higher with respect to other reports [15], [26], probably due to the extension of the scan length.

With respect to effective radiation doses, there is a wide disparity in reported values for a single coronary calcium screening, although there is a general agreement on average values. Kim *et al.* reported median and mean values of 2.3 and 3.1 mSv (range 0.8–10.5), respectively [27]. The AHA Science Advisory reported representative values of 3 mSv (range 1–12 mSv) [28]. Recent recommendations suggest the average value to remain in the range of 1.0–1.5 mSv and should not exceed 3.0 mSv [29]. Our dose values did not exceed 2.1 mSv, remaining below average thresholds reported values using the same scanner model [27].

This study has some limitations that need to be addressed. The cross-sectional design of our study did not allow assessment of time-dependent and causal relationships between parameters. Recent evidence regarding the lack of TAC screening to improve cardiovascular risk prediction beyond Framingham Risk Score and CAC screening are based on standard TA evaluation [5]. The incorporation of “hidden” aortic regions has shown that it may be worth discussing how lack of consistency in field of measurement settings may have limited the prognostic value of TAC in previous prospective studies. There are two different mechanisms of vascular calcification: intimal (atherosclerotic) and medial (arteriosclerotic) [30]. Whereas CAC is mostly intimal, TAC is thought to better represent a generalized atherosclerosis process [4], [7]. The differentiation of the aortic wall from the lumen would need enhanced CT protocols that are not recommended for our patients [31]. Aortic valve calcium was not measured in this study because the vessel segmentation method was limited to tubular cross-sections starting at the left main coronary artery [8]. Our patients were mostly men due to a recruitment bias. Finally, TA geometry was accurately estimated in 3D but size and shape variables were not associated with TAC distribution. The association between aortic morphology, calcifications and traditional risk factors (i.e. hypertension) should be addressed in future investigations.

In conclusion, calcium screening in the TA revealed that the aortic arch and the proximal descending aorta, hidden in standard TA evaluations, concentrated most of the calcifications, being the arch the most densely affected segment per cm length. In our population of asymptomatic subjects at increased cardiovascular risk, TAC prevalence doubled from 31% to 64% using the extended scan method. Middle-aged women were more prone to have calcifications in those hidden portions and became candidates for reclassification. Our results should encourage prospective studies to investigate whether extended TAC assessment improves cardiovascular risk prediction beyond standard methods.
